# Supplementary material for: Optomechanical interface for probing matter-wave coherence
Source: Sci Rep. 2013 Nov 29;3:3378. doi: 10.1038/srep03378 (PMC3843315; doi:10.1038/srep03378)
Supplement: Supplementary Information — for: Optomechanical interface for probing matter-wave coherence [file srep03378-s1.pdf]

Supplementary information for: Optomechanical interface for probing matter-wave coherence

André Xuereb,[1, 2, *] Hendrik Ulbricht,[3] and Mauro Paternostro[1]

[1] *Centre for Theoretical Atomic, Molecular and Optical Physics, School of Mathematics and Physics, Queen's University Belfast, Belfast BT7 1NN, United Kingdom*
[2] *Department of Physics, University of Malta, Msida MSD 2080, Malta*
[3] *School of Physics and Astronomy, University of Southampton, Southampton SO17 1BJ, United Kingdom*

(Dated: October 25, 2013)

S.I. MODELING A STREAM OF PARTICLES

The action of the biprisms in the setup sketched in the main text is similar to that of a balanced beamsplitter on an optical field. Thus, after interacting with the biprism, a single incoming particle can be described through the superposition state

$$\frac{1}{\sqrt{2}}\big(\lvert 0,1\rangle_{\text{part}}+\lvert 1,0\rangle_{\text{part}}\big)=\sum_{r=0}^{1}\binom{1}{r}\lvert 1-r,r\rangle_{\text{part}}\,. \tag{S.1}$$

Here we will show that the state of a stream of N particles after traversing the biprism can be written as

$$\lvert\psi\rangle_{\text{part}}\equiv\sum_{r=0}^{N}\binom{N}{r}\lvert N-r,r\rangle_{\text{part}}\,, \tag{S.2}$$

assuming that the total time separating the arrival of the first and last particles is very small compared to all the other timescales of the problem (as per the discussion in the main text). Suppose we fire two bursts of N_1 and N_2 particles at the two mirrors, with the time separation between the two bursts being negligible compared to τ_{m}. Then, the particle state after the biprism reads

$$\begin{aligned}\lvert\psi\rangle_{\text{part}}&=\sum_{r_1=0}^{N_1}\sum_{r_2=0}^{N_2}\binom{N_1}{r_1}\binom{N_2}{r_2}\lvert(N_1+N_2)-(r_1+r_2),(r_1+r_2)\rangle_{\text{part}}\\&=\sum_{r=0}^{N}\sum_{\substack{r'=-N_2,\\(r\pm r')\text{ even}}}^{N_1}\binom{N_1}{(r+r')/2}\binom{N_2}{(r-r')/2}\lvert N-r,r\rangle_{\text{part}}\,,\end{aligned} \tag{S.3}$$

where $N=N_1+N_2$. Next, we use the fact that

$$\binom{n}{k}=0 \tag{S.4}$$

whenever $k<0$ or $k>n$, as follows from the definition of the Euler Gamma function, which diverges for non-positive integer values. This fact allows us to rewrite the sum above as

$$\sum_{\substack{r'=-N_2,\\(r\pm r')\text{ even}}}^{N_1}\cdots\quad\equiv\sum_{\substack{r'=-r,\\(r\pm r')\text{ even}}}^{r}\cdots\,, \tag{S.5}$$

since $r'>N_1$ implies that the only terms that might contribute require $r\geq r'>N_1$, such that $(r+r')/2>N_1$; i.e., every such term is zero. Similarly, terms with $r'<-N_2$ do not contribute. It does not change anything, therefore,

* Email address: andre.xuereb@gmail.com

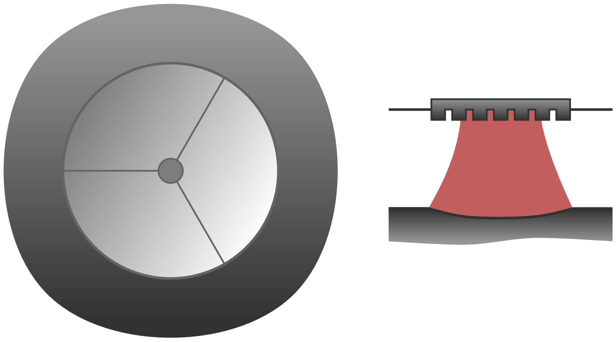

FIG. S.1. Example mirror structure having properties that are compatible with the requirements of our thought experiment. The figure on the left shows a plan of the mirror, with the large (immobile) cavity mirror in the background. On the right, we show a section of the optical mode inside the microcavity; the mobile mirror may be patterned [S2] in order to increase its reflectivity.

to extend the lower (upper) limit of the sum to $-r$ (r) when $r > N_2$ ($r > N_1$). Moreover, the requirement for the bottom factor of the binomials to be non-negative requires $|r'| \le r$. Therefore, the lower (upper) limits of the sum may be changed to $-r$ (r) in any case. Let us now identify $n \equiv N_1$, $m \equiv N_2$, $k \equiv (r + r')/2$, and $p \equiv r$, all of which are non-negative integers. From Ref. [S1, §0.156 Eq. (1)] we know that

$$\sum_{k=0}^{p} \binom{n}{k} \binom{m}{p-k} \equiv \binom{n+m}{p}, \tag{S.6}$$

that is,

$$\sum_{\frac{r+r'}{2}=0}^{r} \binom{N_1}{(r+r')/2} \binom{N_1}{(r-r')/2} \equiv \binom{N_1+N_2}{r} \equiv \binom{N}{r}, \tag{S.7}$$

where the sum runs over the values of r' for which $r + r'$ is even, since k must be an integer. Moreover, $0 \le k \le p$, which is equivalent to $-r \le r' \le r$. Thus, we can rewrite

$$\sum_{\frac{r+r'}{2}=0}^{r} \cdots \quad \equiv \sum_{\substack{r'=-r,\\ (r\pm r')\ \mathrm{even}}}^{r} \cdots, \tag{S.8}$$

which gives Eq. (S.2). In other words, proceeding by induction, multiple ‘bursts’ can be treated *identically* to a single burst with the same total number of particles.

S.II. EFFECT OF COLLISIONS ON MIRROR

The estimates in the main text resulted from calculations using structure shown in Fig. S.1. In particular, we suppose that the central (reflective) part of the suspended mirror is circular with diameter 10 µm and thickness 500 nm, and made from silicon nitride (yielding a mass of 1.3×10^{-13} kg for the mirror itself). The three mirror supports are assumed to be robust enough for nonlinear effects to be ignored. The fundamental motion of such a system, to a good approximation, involves the circular mirror itself undergoing centre-of-mass oscillations. A rudimentary finite element analysis of this structure shows that at the start of the harmonic cycle, only the fundamental mode carries a significant amount of net forward momentum. This leads us to postulate that upon collision, whereas only a small fraction of the energy of the particle is transferred to the fundamental mode essentially all of the momentum is. This argument relies on the mirror not being punctured by the particles; we follow Figs. 3 and 4 in Ref. [S3] as an approximation to our situation and determine that a thickness of 500 nm should be enough to stop the incident ions. The collision time stated in the main text is an approximated upper bound, determined as the time it would take for the particles to come to a complete stop over a distance of 500 nm. The rest of the energy imparted to the mirror through the collision process goes to excite higher-order modes, most of which are coupled to the optical fields significantly more weakly than the fundamental mode. Insofar as the optical readout process of the fundamental mechanical mode happens on a faster timescale than the decoherence timescale relevant to these mechanical modes, as explained in the main text, the excitation of these modes should not affect the results reported in the main text.

S.III. POST-COLLISION DENSITY MATRIX

We begin recalling the form of the particle state $|\psi\rangle_{\rm part} = \binom{2N}{N}^{-1/2} \sum_{n=0}^{N} \binom{N}{n} |N-n, n\rangle_{\rm part}$. The density matrix $|\psi\rangle\langle\psi|_{\rm part}$ corresponding to this state can be written as

$$\rho_{\rm part}(0^-) = \left[\sum_{r=0}^{N} \binom{N}{r}^2\right]^{-1} \sum_{r_1,r_2=0}^{N} \binom{N}{r_1}\binom{N}{r_2} \Phi(r_1-r_2) |N-r_1, r_1\rangle\langle N-r_2, r_2|_{\rm part}\,, \tag{S.9}$$

where the function $\Phi(r)$ acts as a control that changes $\rho_{\rm part}(0^-)$ from a coherent superposition [$\Phi(r)=1$] to an incoherent mixture [$\Phi(r)=\delta_{r,0}$, where $\delta_{i,j}$ is the Kronecker delta]. Combining this with the density matrix

$$\rho_{\rm mech}(0^-) = \frac{1}{\pi^2\bar{n}^2} \iint {\rm d}^2\beta_1 {\rm d}^2\beta_2\, e^{-\frac{|\beta_1|^2+|\beta_2|^2}{\bar{n}}} |\beta_1,\beta_2\rangle\,\langle\beta_1,\beta_2|_{\rm mech}\,,$$

expressed in terms of joint coherent states, and where the mirrors are taken to be in thermal states with average phonon occupation $\bar{n}$ (assumed to be the same for both mirrors; whilst this assumption is not crucial for our proposal, it allows us to simplify our formal treatment of the problem), we obtain the density matrix for the total system just before the collision occurs

$$\begin{aligned}\rho(0^-) = \rho(0^-)_{\rm part}\otimes\rho(0^-)_{\rm mech} = \frac{1}{\pi^2\bar{n}^2\sum_{r=0}^{N}\binom{N}{r}^2} \sum_{r_1,r_2=0}^{N} \binom{N}{r_1}\binom{N}{r_2}\Phi(r_1-r_2) \\ \times \iint {\rm d}^2\beta_1\, {\rm d}^2\beta_2\, e^{-(|\beta_1|^2+|\beta_2|^2)/\bar{n}} |N-r_1,r_1\rangle|\beta_1,\beta_2\rangle\langle\beta_1,\beta_2|\langle N-r_2,r_2|\,.\end{aligned} \tag{S.10}$$

We model the collision process as described in the main text, yielding the normalised post-collision joint mechanical density matrix

$$\begin{aligned}\rho_{\rm mech}(0^+) = \sum_{r_1,r_2=0}^{N} \binom{N}{r_1}\binom{N}{r_2} \frac{\Phi(r_1-r_2)}{\pi^2\bar{n}^2\mathcal{Q}} \iint {\rm d}^2\beta_1\,{\rm d}^2\beta_2\, e^{-\frac{|\beta_1|^2+|\beta_2|^2}{\bar{n}}} e^{-i\gamma(r_1-r_2)\,{\rm Re}\{\beta_1-\beta_2\}} \\ \times |\beta_1+i\gamma(N-r_1), \beta_2+i\gamma r_1\rangle\langle\beta_1+i\gamma(N-r_2), \beta_2+i\gamma r_2|\,,\end{aligned} \tag{S.11}$$

where $\mathcal{Q} = \sum_{r_1,r_2=0}^{N}\binom{N}{r_1}\binom{N}{r_2}\Phi(r_1-r_2)e^{-(2\bar{n}+1)\gamma^2(r_1-r_2)^2} = \sum_{r=-N}^{N}\binom{2N}{N+r}\Phi(r)e^{-(2\bar{n}+1)\gamma^2 r^2}$. The incoherent case is strikingly different, and we find $\mathcal{Q}=2^{2N}$.

S.IV. WIGNER–WEYL QUASIPROBABILITY DISTRIBUTION

Describing our system in phase space makes subsequent calculations less cumbersome, so we choose to calculate the Wigner–Weyl quasiprobability distribution that corresponds to $\rho_{\rm mech}(0^+)$ derived above. To do so, we take the two-dimensional complex Fourier transform of the characteristic function of the state [S4, Eqs. (3.3.6) and (3.3.7)]:

$$W(\beta_1,\beta_2) = \frac{1}{\pi^4}\iint {\rm d}^2 b_1\,{\rm d}^2 b_2\, e^{-2i\,{\rm Re}\{\beta_1 b_1^* + \beta_2 b_2^*\}}\chi(b_1,b_2)\,, \tag{S.12}$$

where $\chi(b_1,b_2) = {\rm Tr}\{\hat{D}_1(ib_1)\hat{D}_2(ib_2)\rho_{\rm mech}(0^+)\}$. Here, $\hat{D}_i(\alpha)$ $(i=1,2)$ is the Weyl displacement operator introduced in the main text, operating on the mechanical state of mirror. A long but otherwise straightforward calculation yields

$$\begin{aligned}W(\beta_1,\beta_2) = \sum_{r_1,r_2=0}^{N}\binom{N}{r_1}\binom{N}{r_2} \frac{\Phi(r_1-r_2)e^{-\frac{2}{2\bar{n}+1}\left[|\beta_1-\frac{i}{2}\gamma(2N-r_1-r_2)|^2+|\beta_2-\frac{i}{2}\gamma(r_1+r_2)|^2\right]}}{\pi^2\left(\bar{n}+\frac{1}{2}\right)^2\mathcal{Q}} \\ \times\cos\bigl[2\gamma(r_1-r_2)\,{\rm Re}\{\beta_1-\beta_2\}\bigr].\end{aligned} \tag{S.13}$$

This Wigner function is essentially a sum of Gaussian terms modulated by an interference factor (which is always equal to 1 for the ‘classical’ case). It can be written in a more symmetrical fashion by shifting it as $W_{\rm S}(\beta_1,\beta_2) \equiv$

$W(\beta_1 + \frac{i}{2}\gamma N, \beta_2 + \frac{i}{2}\gamma N)$, whereby

$$W_{\rm S}(\beta_1,\beta_2) = \sum_{r_1,r_2=0}^{N} \binom{N}{r_1}\binom{N}{r_2} \frac{\Phi(r_1-r_2) e^{-\frac{2}{2\bar{n}+1}\left[|\beta_1 - \frac{i}{2}\gamma(N-r_1-r_2)|^2 + |\beta_2 + \frac{i}{2}\gamma(N-r_1-r_2)|^2\right]}}{\pi^2\left(\bar{n}+\frac{1}{2}\right)^2 \mathcal{Q}} \times \cos\left[2\gamma(r_1-r_2)\,\mathrm{Re}\{\beta_1-\beta_2\}\right]. \quad (S.14)$$

Let us recall that a Wigner function with at least one pair of coordinates (β_1, β_2) such that $W(\beta_1, \beta_2) < 0$ does not have a classical explanation and necessarily describes a non-classical state. The measure of non-classicality used throughout the main text [S5] is based on this observation, as discussed in Ref. [S5]

S.V. JOINT HOMODYNE DETECTION

Let us suppose we can read out any quadrature of the mechanical oscillator state through the cavity field. We would like to calculate the possibility of obtaining a joint measurement with amplitudes x and y and phases θ and ϕ. Instead of reading out the individual cavity fields, however, we interfere them on a beamsplitter prior to homodyning. The beamsplitter operator, which we represent by $\hat{B}$ and define in the main text, effectively acts on the mechanical fields in the coherent representation as

$$\hat{B}|\beta_1,\beta_2\rangle = |(\beta_1 + i\beta_2)/\sqrt{2}, (i\beta_1+\beta_2)/\sqrt{2}\rangle\,. \quad (S.15)$$

After operation by the beamsplitter, the state is projected onto the quadratures x_θ and y_ϕ, and the absolute-squared value of the projection measured

$$P(x_\theta, y_\phi) = |\langle x_\theta, y_\phi|\hat{B}\,\rho_{\rm mech}(0^+)\,\hat{B}^\dagger|x_\theta,y_\phi\rangle|^2\,. \quad (S.16)$$

It is convenient to define the quantities

$$\bar{x}_{\theta,r_1,r_2} = \tfrac{1}{2}\gamma\left[(2N-r_1-r_2)\sin\theta - (r_1+r_2)\cos\theta - i(2\bar{n}+1)(r_1-r_2)(\cos\theta-\sin\theta)\right], \quad (S.17)$$

$$\bar{y}_{\phi,r_1,r_2} = \tfrac{1}{2}\gamma\left[(r_1+r_2)\sin\phi - (2N-r_1-r_2)\cos\phi + i(2\bar{n}+1)(r_1-r_2)(\cos\phi-\sin\phi)\right], \quad (S.18)$$

so that the probability distribution in Eq. (S.16) becomes

$$P(x_\theta,y_\phi) = \frac{1}{\pi^2\left(\bar{n}+\frac{1}{2}\right)^2\mathcal{Q}^2}\left|\sum_{r_1,r_2=0}^{N}\binom{N}{r_1}\binom{N}{r_2}\Phi(r_1-r_2)e^{-\frac{1}{2\bar{n}+1}\left[(x_\theta-\bar{x}_{\theta,r_1,r_2})^2+(y_\phi-\bar{y}_{\phi,r_1,r_2})^2\right]}e^{-(2\bar{n}+1)\gamma^2(r_1-r_2)^2}\right|^2. \quad (S.19)$$

This expression is not quite the square of a sum of Gaussians because of the imaginary parts of $\bar{x}_{\theta,r_1,r_2}$ and $\bar{y}_{\phi,r_1,r_2}$ that, for the coherent case, lead to interference effects that are completely absent from the 'classical' one. Indeed, if we decompose $\bar{x}_{\theta,r_1,r_2} = \bar{x}^{\rm r}_{\theta,r_1,r_2} + i\bar{x}^{\rm i}_{\theta,r_1,r_2}$, and $\bar{y}_{\phi,r_1,r_2} = \bar{y}^{\rm r}_{\phi,r_1,r_2} + i\bar{y}^{\rm i}_{\phi,r_1,r_2}$ we can rewrite this expression as

$$P(x_\theta,y_\phi) = \frac{1}{\pi^2\left(\bar{n}+\frac{1}{2}\right)^2\mathcal{Q}^2}\left|\sum_{r_1,r_2=0}^{N}\binom{N}{r_1}\binom{N}{r_2}\Phi(r_1-r_2)e^{-\frac{1}{2\bar{n}+1}\left[(x_\theta-\bar{x}^{\rm r}_{\theta,r_1,r_2})^2+(y_\phi-\bar{y}^{\rm r}_{\phi,r_1,r_2})^2\right]} \times e^{i\sqrt{2}\gamma(r_1-r_2)\left[\cos\left(\theta+\frac{\pi}{4}\right)(x_\theta-\bar{x}^{\rm r}_{\theta,r_1,r_2})-\cos\left(\phi+\frac{\pi}{4}\right)(y_\phi-\bar{y}^{\rm r}_{\phi,r_1,r_2})\right]} \times e^{-\left(\bar{n}+\frac{1}{2}\right)\gamma^2(r_1-r_2)^2\left[\sin^2\left(\theta+\frac{\pi}{4}\right)+\sin^2\left(\phi+\frac{\pi}{4}\right)\right]}\right|^2. \quad (S.20)$$

A. Behaviour of resonance with number of particles

Consider now $\mathrm{Re}\{\bar{x}_{\theta,r_1,r_2}\} = \frac{1}{2}\gamma\left[(r_1+r_2)\sin\phi - (2N-r_1-r_2)\cos\phi\right]$, which is independent of r_1 and r_2 for $\sin\theta = -\cos\theta = \pm\frac{1}{\sqrt{2}}$. Let us further choose to monitor the coincidences for which $x = y$. In this case, when $\phi = -\theta - \frac{\pi}{2}$, we have $\mathrm{Re}\{\bar{x}_{\theta,r_1,r_2}\} = \mathrm{Re}\{\bar{y}_{\phi,r_1,r_2}\}$, and at $\theta = \theta_0 = -\frac{\pi}{4} + 2n\pi$ $(n \in \mathbb{N})$, all these curves (seen as

functions of θ or ϕ) coincide. At this point one observes interference effects in the quantum-mechanical case. After some algebra, we get to

$$P(x_\theta, y_\phi) = \begin{cases} \frac{1}{\pi^2(2\bar{n}+1)^2} & \text{(incoherent case)}, \\ \frac{2^{4N}}{\pi^2(2\bar{n}+1)^2}\left[\sum_{r=-N}^{N}\binom{2N}{N+r}e^{-(2\bar{n}+1)\gamma^2 r^2}\right]^{-2} & \text{(coherent case)}. \end{cases} \tag{S.21}$$

The former of these equations is *independent of the number of particles or the strength of the interaction*, whereas the latter is approximately linear in N. Indeed, for N and γ or $\bar{n}$ large enough we can write

$$P(x_\theta, y_\phi) = \begin{cases} \frac{1}{\pi^2(2\bar{n}+1)^2} & \text{(incoherent case)}, \\ \frac{N}{\pi(2\bar{n}+1)^2} & \text{(coherent case)}. \end{cases} \tag{S.22}$$

B. Effect of homodyning angle on the resonance peak

With the choice of parameters analysed above, $P(x_\theta, y_\phi)$ has Gaussian peaks for both coherent and the incoherent cases. Let us now set $\theta = \theta_0 + \nu$ and $\phi = -\theta_0 - \frac{\pi}{2} + \nu$. It can be shown that, to lowest order in ν, we have

$$P(x_\theta, y_\phi) = \begin{cases} \frac{1}{\pi^2(2\bar{n}+1)^2} & \text{(incoherent case)}, \\ \frac{1}{\pi^2(2\bar{n}+1)^2}\frac{\left|\sum_{r_1,r_2=0}^{N}\binom{N}{r_1}\binom{N}{r_2}e^{-2i\gamma^2(N-r_1-r_2)(r_1-r_2)\nu}\right|^2}{\left[\sum_{r=-N}^{N}\binom{2N}{N+r}e^{-(2\bar{n}+1)\gamma^2 r^2}\right]^{-2}} & \text{(coherent case)}. \end{cases} \tag{S.23}$$

There is again a qualitative difference between the two cases: The peak in the incoherent case is constant with respect to small variations in ν, whereas the coherent case exhibits characteristic variations with such parameter.

S.VI. MOMENTUM IMPARTED BY A COLLIDING PARTICLE ONTO A MIRROR

Let us work in a classical picture to estimate the effect of the adsorption of a single particle on a mirror. The change in (dimensionless) momentum of the mirror, Δp, due to the instantaneous adsorption of a particle travelling with momentum $p_{\rm part}$, is given by $M\omega_{\rm m}x_{\rm zpt}\Delta p = -p_{\rm part}$. Here M is the mass of the mirror and $\omega_{\rm m}$ its oscillation frequency. We can write this as $\Delta p = -G$ with

$$G = 2\pi \times \frac{x_{\rm zpt}}{\lambda_{\rm dB}} = \frac{p_{\rm part}}{\sqrt{\hbar M \omega_{\rm m}}}, \tag{S.24}$$

where $\lambda_{\rm dB} = h/p_{\rm part}$ is the de Broglie wavelength of the matter wave. The extent of the zero-point fluctuations of the mirror motion is $x_{\rm zpt} = \sqrt{\hbar/(M\omega_{\rm m})}$. We divide G by a factor of $\sqrt{2}$, in accordance with the definition of $\hat{D}_{1,2}$, to obtain the parameter γ used in the paper.

S.VII. FREQUENCY SHIFT DUE TO A SINGLE COLLISION

We work in a classical picture to approximate the frequency shift obtained in a resonant cavity as the result of the adsorption of a single particle. The momentum imparted onto the mirror results in a maximal displacement ΔL such that $M\omega_{\rm m}\Delta L = p_{\rm part} \Rightarrow \Delta L = p_{\rm part}/(M\omega_{\rm m})$. For a resonant cavity, we have $L_{\rm c} = \frac{n}{2}\lambda_{\rm c}$, where $n \in \mathbb{N}$. Thus,

$$\Delta L = \tfrac{n}{2}\Delta\lambda \Rightarrow \frac{\Delta L}{L_{\rm c}} = \frac{\Delta\lambda}{\lambda_{\rm c}}\,. \tag{S.25}$$

We can therefore calculate the maximum frequency modulation

$$\omega_{\rm c} = \frac{2\pi c}{\lambda_{\rm c}} \Rightarrow \Delta\omega = \frac{\omega_{\rm c}}{L_{\rm c}}|\Delta L| = \frac{\omega_{\rm c} p_{\rm part}}{L_{\rm c}\omega_{\rm m} M} = 2\pi \times \frac{\omega_{\rm c} x_{\rm zpt}^2}{L_{\rm c}\lambda_{\rm dB}}\,. \tag{S.26}$$

[S1] I. S. Gradshteyn and I. M. Ryzhik, *Table of integrals, series and products*, 5th ed. (Academic Press, 1994).

[S2] U. Kemiktarak, M. Metcalfe, M. Durand, and J. Lawall, Appl. Phys. Lett. **100**, 061124 (2012).
[S3] S. Miyagawa, Y. Ato, and Y. Miyagawa, Jpn. J. Appl. Phys. **23**, 1380 (1984).
[S4] M. O. Scully and M. S. Zubairy, *Quantum Optics*, 1st ed. (Cambridge University Press, 1997).
[S5] A. Kenfack and K. Życzkowski, J. Opt. B **6**, 396 (2004).
